# Rapid Reversal of Forearm Supinator Rigidity With Topical Isomerized Potassium Linoleate: A Novel Perspective on Microbiome-Induced Tetany

**DOI:** 10.7759/cureus.80896

**Published:** 2025-03-20

**Authors:** David G Changaris

**Affiliations:** 1 Outpatient Department, Independent Researcher, Louisville, USA

**Keywords:** central vertigo, extended wrist rotation, isomerized potassium linoleate, malassezia, parkinsonism, parkinson's disease, quorum sensing, rigidity, tetany, vertigo of central origin

## Abstract

Microbes can alter host behavior, immunity, and neurological function at a distance without extension into the brain and spinal cord. Clostridia provides a predicate for such an infection in the periphery by causing “lock jaw” and generalized tetany. This case series presents five patients who showed rigidity or tetany of the forearm. All were diagnosed with vertigo of central origin by video nystagmography (VNG) and posturography. Each had an apparent slow-rolling tetany, most visible in the forearm. Each had a consistent focus of pain within the supinator, diminution of extended wrist rotation, and tender, taut bands. None had clinical evidence of injury to the ulnar, radial, or median nerves, ulnar epicondyles, or wrist. The author applied a commercial preparation of a cleanser containing isomerized potassium linoleate (KCLA) to the skin overlying the forearm's biceps, supinator, and pronator as an “alternative” medical approach to refractory rigidity and tenderness. The tenderness resolved within two to four minutes. After 3-10 minutes, follow-on extended wrist rotation improved toward the norm (p < 0.01). The improved range of motion lasted beyond discharge from the clinic visit. The rapid response in this series suggests the commensal skin biome may contribute to clinical tetany in the forearm supinator.

## Introduction

In 2001, the National Institutes of Health (NIH) consensus meeting defined rigidity as a condition in which one muscle contracts while its opposing muscle fails to relax [1]. Clinically, rigidity limits the range of motion, and progressive cases can lead to severe stiffness and tremors. In patients with post-traumatic brain and spinal cord injuries, rigidity and tremors are key diagnostic markers for tracking neurodegeneration. The Unified Parkinson’s Disease Rating Scale (UPDRS) includes wrist rotation assessment [2] as part of the clinical diagnosis of Parkinson’s Disease (PD). Additionally, research has established a link between traumatic brain injuries and neurodegenerative diseases [3,4], such as PD and Alzheimer’s Disease (AD). Rigidity is commonly associated with dysfunction in the central nervous system (CNS), resulting in sustained, involuntary muscle contraction.

For some, the terminology used to document rigidity follows the “Law of the Instrument” [5]. “Taut Bands” become the massage therapist’s phenotypic presentation of rigidity. Rigidity itself is non-specific and stems from diverse etiologies. For instance, rigidity due to infectious myositis necessitates antimicrobial intervention, whereas myofascial pain syndrome and taut bands may respond to manual therapy, electrolyte correction, or pressure release. Keeping the term “rigidity” neutral to implied treatment or causation remains challenging. The treating professional uses a synonym for rigidity linked to a specific treatment. This constrains the clinician’s dialogue as to the “source” of the rigidity. From this perspective, tetany is rigidity caused by *Clostridium tetani*. Both tetany and rigidity mean sustained, involuntary muscle contraction.

Consider the symptoms and signs of rigidity caused by clostridial infection as a predicate for rigidity or tetany caused by a distant infection. For example, once in the body through a distal puncture wound, *C. tetani* releases “tetanus toxin,” causing generalized tetany and “lockjaw.” Untreated, without prior immune augmentation, tetanus toxin accesses motor neurons and travels retrogradely to the spinal cord and brain. The toxin, binding gangliosides, disrupts inhibitory neurotransmission throughout the nervous system, resulting in involuntary muscle contractions and generalized rigidity. Tetanus highlights the profound influence of peripheral microbial infection on CNS function. It still leads to severe and fatal outcomes [6].

Fungi can initiate complex behavioral changes within their host without infecting the CNS [7]. Fatal microbial parasitism occurs within tropical ants, induced by *Ophiocordyceps*. This fungus influences complex behavior, including jaw spasms, without physical extension into the CNS [8,9].

Relevant to this setting, Quorum Sensing Agents (QSAs) can exert remote primary effects on the mammalian immune system and disrupt mammalian brain activity [10]. Fungal and bacterial microbes secrete species-specific chemicals that allow microbial sensing of the local density. Within the emerging Quorum Sensing paradigm, microbes initiate new invasive behaviors upon reaching a sensed density. Many QSAs are homoserine lactones. Upon reaching a quorum or density of a specific lactone, *Pseudomonas* initiates collective “biofilm” formation [11]. Biofilm formation offers formidable protection against human antimicrobial treatment. Efforts to pharmacologically alter microbial quorum-sensing behavior offer hope for antibiotics' waning efficacy.

## Case presentation

Each case was examined with the extended wrist rotation by the “Handshake” method [2]. The case extends the arm while the examiner holds the patient’s elbow with one hand, while holding the hand as a handshake. The examiner instructs the case to point his thumb to the ceiling and keep the elbow crease pointing to the ceiling. With the initiation of rotation of the elbow crease, involuntary rotation occurs, and the effective rotation is measured. The external and internal rotations represent the supinator and pronator muscles, respectively. Each case showed tenderness within the supinator muscle. Some also had tenderness in the pronator muscle. Each had 10 cc of body wash containing a proprietary blend of isomerized potassium linoleate (KCLA; Ceela Naturals, Louisville, KY, USA) applied to the skin overlying the medial lower half of the biceps and to the insertions of both the pronator and supinator muscles within the forearm. The “slip” of the gel allowed gentle massaging of the pronator and supinator intramuscular bands. The massage of the muscle continued for one to three minutes. Each case, within this period, showed a clear palpable loss of muscle tone and softening of the intramuscular cords.

Post-traumatic cases were followed for persisting vertigo of central origin, defined by video nystagmography (VNG) and posturography (MedTrak VNG 2012; MedTrak VNG, Inc., Henderson, NV, USA). Each had a significant traumatic event. The VNG showed a saccadic delay at or exceeding 350 milliseconds. Both analysis of variance (ANOVA) and paired t-test were employed to identify the significance of the change in the wrist rotation on the treatment effect of KCLA.

Case 1

This 37-year-old female, two years after her motor vehicular accident, complained of writing difficulty. Her hand would cramp as she wrote notes with a pen. The palpation of her supinator showed marked tenderness, along with 0.5-1 cm cords extending from just below the elbow to the insertion on the radius. Her pronator was minimally tender. Table 1 contains her extended wrist rotation measures.

**Table 1:** Raw Data From Each Case Before and After Treatment The supination or external wrist rotation has a greater impairment initially than the pronation or internal wrist rotation. Applying isomerized potassium linoleate (KCLA) to the skin overlying these muscles results in the softening of the muscles and improves range of motion in both directions. Simply looking at the raw data points to the need for further study. The data may be helpful to someone making an estimate of power for sample size when designing a study. While the data technically shows statistical significance, the inherent sample bias of a case series makes it impossible to define clinical significance.

Case	Right External Wrist Rotation	Right Internal Wrist Rotation	Post Treatment: External Wrist Rotation	Post Treatment: Internal Wrist Rotation
1	40	60	70	70
2	30	50	80	80
3	40	60	70	85
4	30	60	80	80
5	30	30	80	80

**Figure 1:**
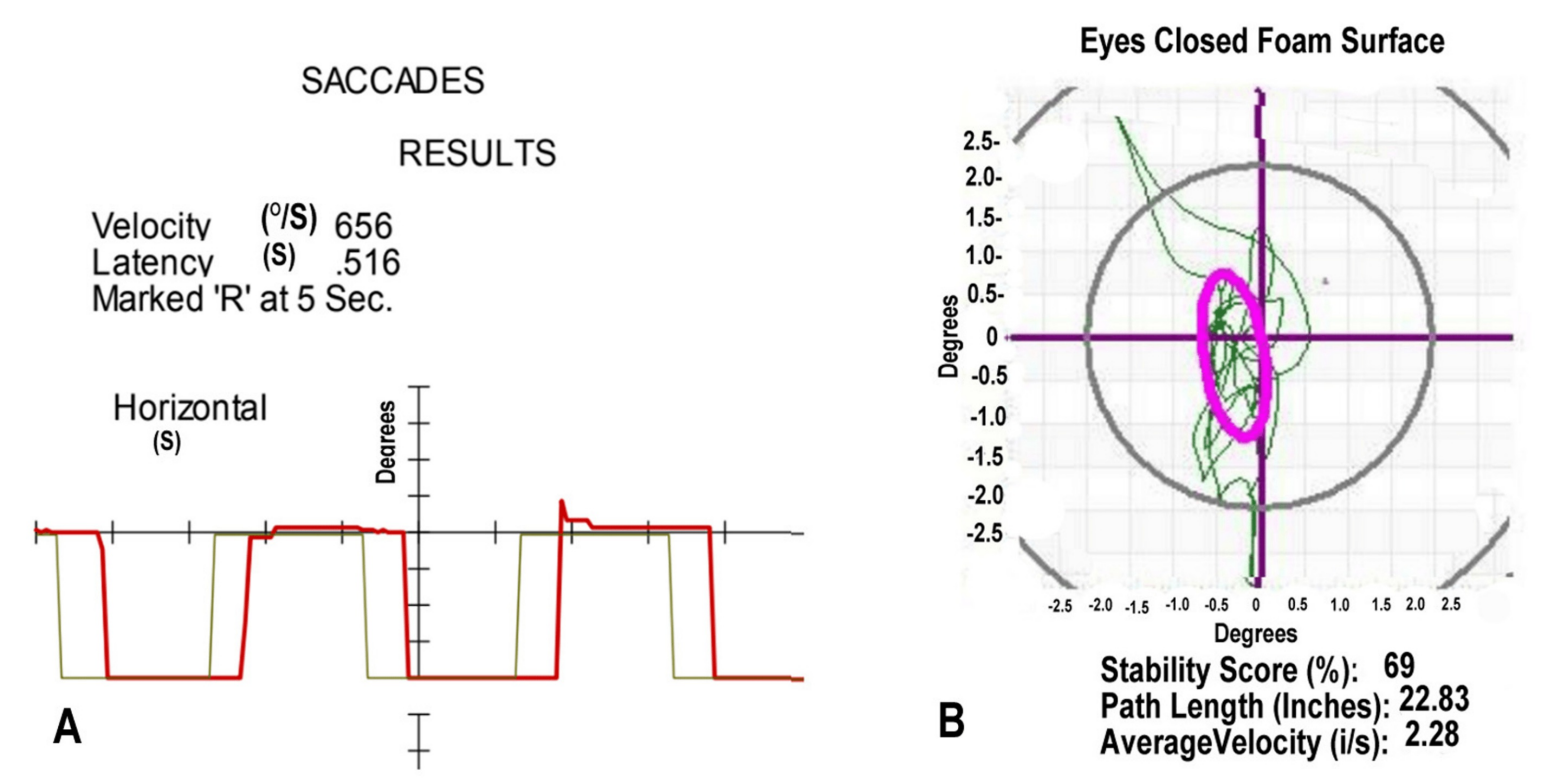
Video Nystagmography and Computerized Posturography for Case 1 (A) The patient's saccades (red line), measured with a moving light, show a latency of 0.516 seconds (S); normal ranges to 350 milliseconds. The 656 degrees/second (°/S) velocity of the saccade is normal. (B) Head movement (green line) during posturography shows measures (degrees) below the third standard deviation from the norm while standing on a foam surface with eyes closed. The purple oval defines the normal range.

Her initial VNG showed delayed saccades in Figure 1A. Her posturography showed abnormal head movement in Figure 1B. Her initial diagnoses included vertigo of central origin, a disorder of the brain's vestibular system. She actively participated in balance training, with a temporary resolution of her vertigo.

Case 2

**Figure 2:**
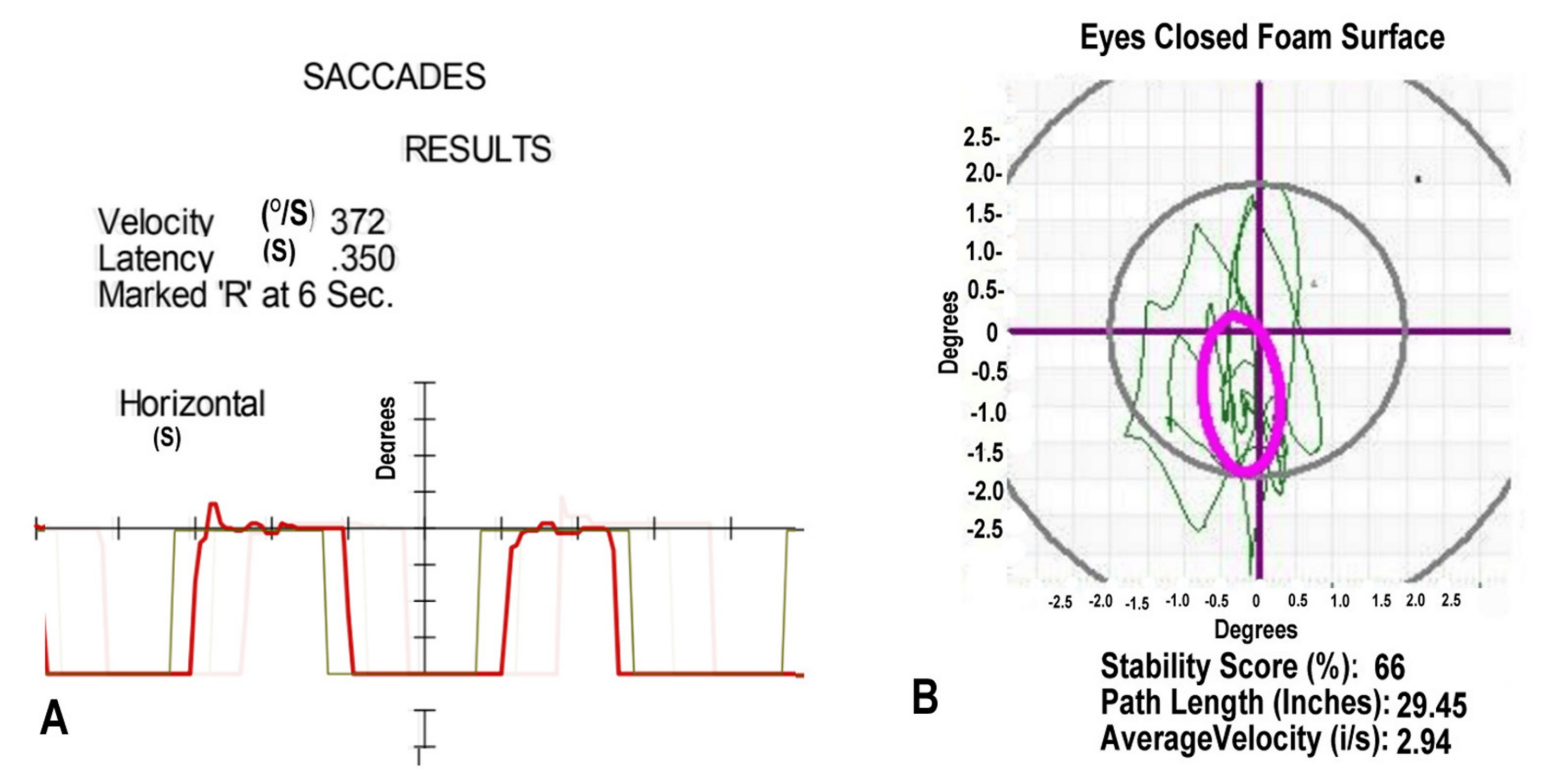
Video Nystagmography and Computerized Posturography for Case 2 (A) Saccades measured with a moving light. The patient (red line) shows a latency of 0.350 seconds; normal ranges to 350 milliseconds. (B) Abnormal head movement (green line) during posturography shows measures below the third standard deviation from the norm while standing on a foam surface with eyes closed. The purple oval defines the normal range.

During a routine follow-up for a motor vehicular accident at one year, this 67-year-old male showed loss of his extended wrist rotation, as recorded in Table 1. He also demonstrated tender 0.5-1 cm diameter, rope-like cords in his supinator. His pronator was minimally tender, without cords. Within the workup one year prior, he showed deficits during his posturography. His VNG showed delayed saccades in Figure 2A. Posturography showed abnormal head movement in Figure 2B. These findings were consistent with vertigo of central origin. Treatment included balance rehabilitation, becoming asymptomatic with his vertigo.

Case 3

Having completed a course of balance therapy, this 54-year-old male presented two months post motor-vehicular accident. He showed a loss of range of motion in his extended wrist rotation. His supinator contained tender 0.5-1 cm cords extending below the elbow to its insertion on the radius. His pronator was minimally tender. His extended wrist rotation showed a loss of range of motion, as recorded in Table 1.

**Figure 3:**
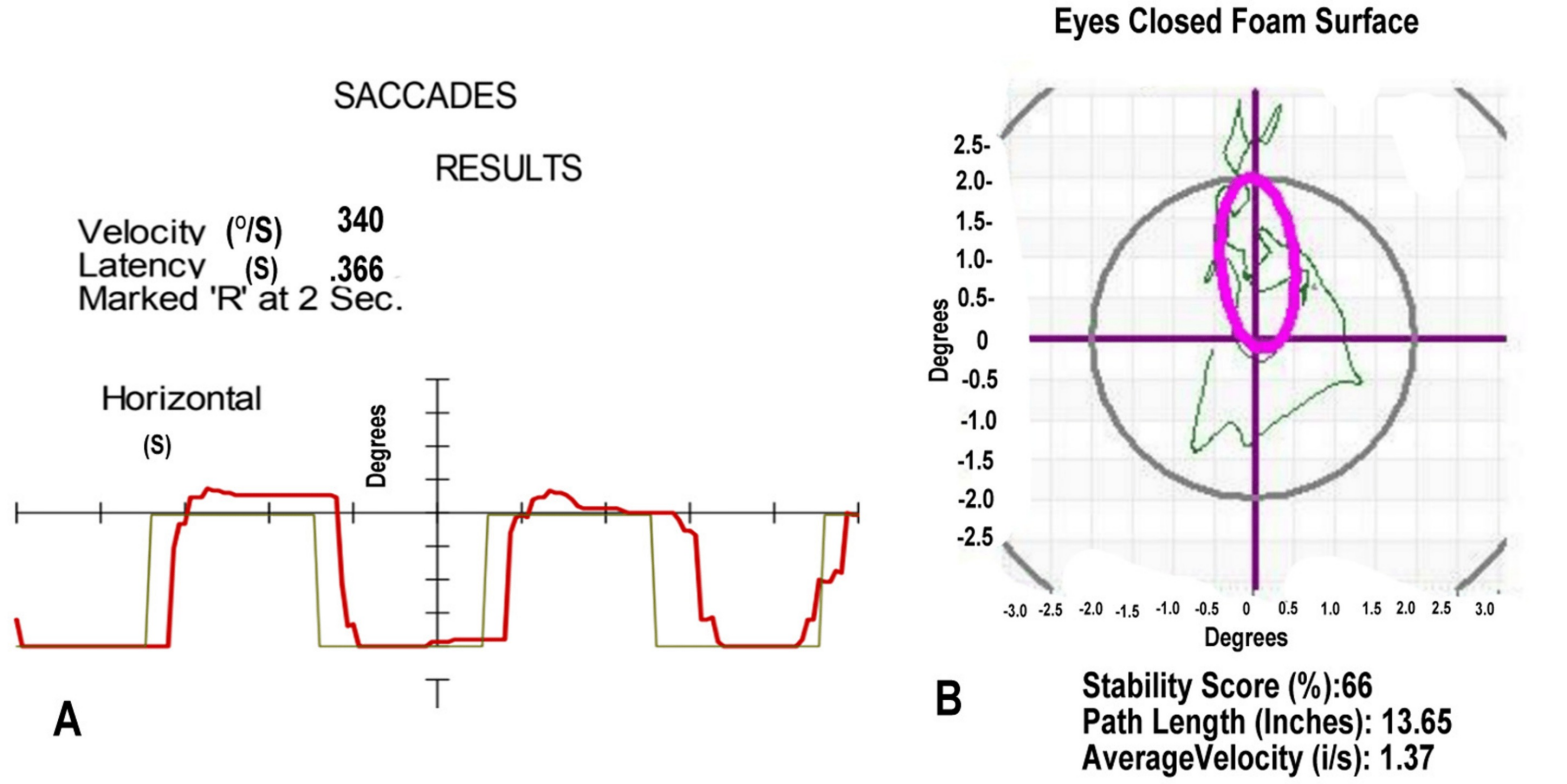
Video Nystagmography and Computerized Posturography for Case 3 (A) The saccades (red line), measured with a moving light, show a slightly prolonged latency of 0.366 seconds. (B) Head movement (green line) during posturography, measuring head movement beyond the norm while standing on a foam surface with eyes closed. The purple oval defines the normal range.

Shortly after his accident, he showed a delay in saccades on VNG, as shown in Figure 3A. He also demonstrated a loss of balance capacity on his posturography, shown in Figure 3B. These findings supported his initial diagnosis of vertigo of central origin.

Case 4

At 10 months post-MVA, this 56-year-old male struggled with continuing loss of shoulder range of motion. His extended wrist rotation showed a loss of range of motion, as reported in Table 1. Palpation of his right supinator caused reports of severe pain. His pronator palpation also caused reports of pain, less than the supinator. Within his supinator, he had tender 0.5-1 cm cords extending from below the elbow to the insertion on the radius.

**Figure 4:**
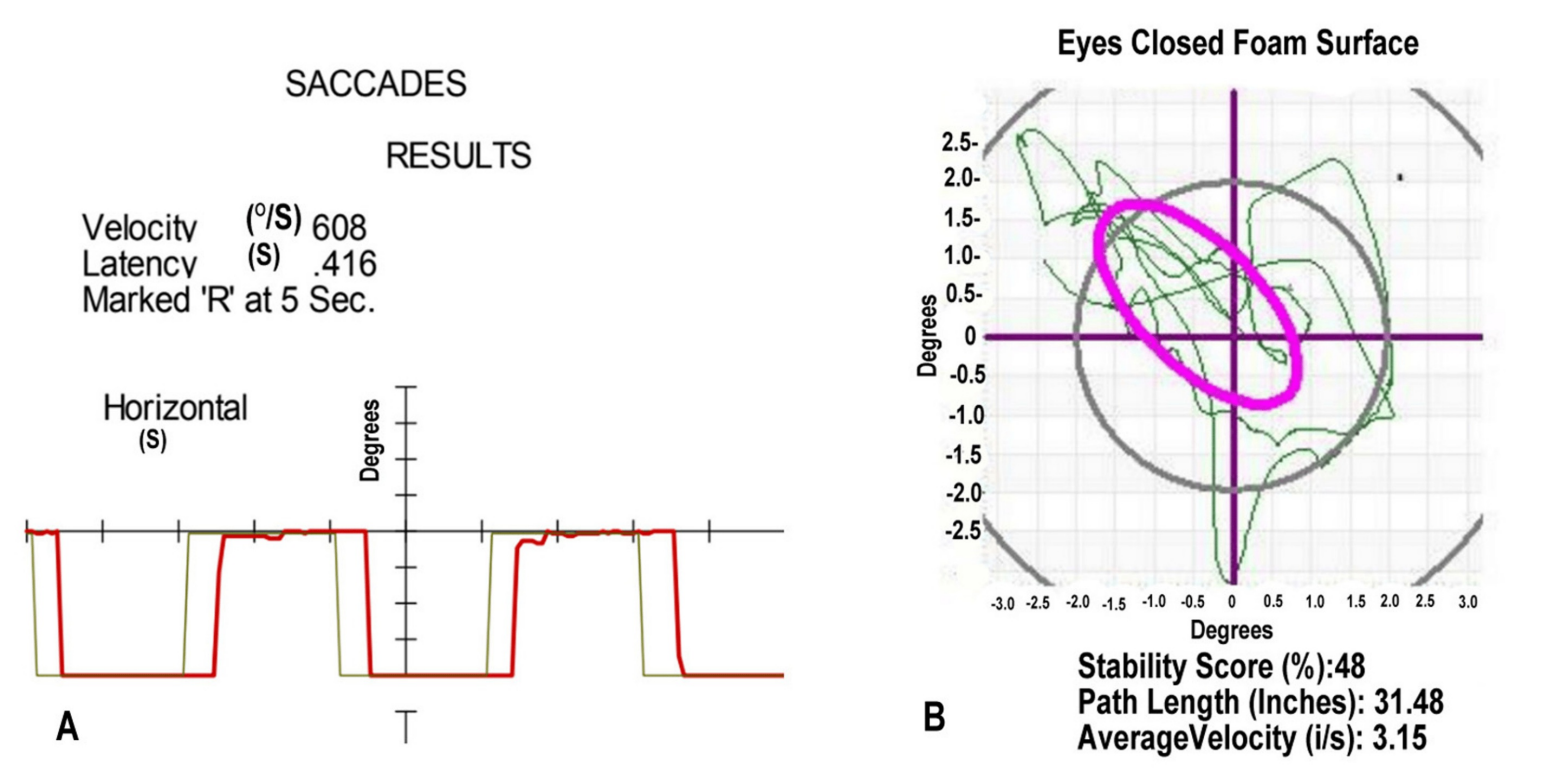
Video Nystagmography and Computerized Posturography for Case 4. (A) The saccades of Case 4, in red line, measured with a moving light, show a prolonged latency of 0.416 seconds. (B) Head movement (green line) during posturography shows measures below the third standard deviation from the norm while standing on a foam surface with eyes closed. The purple oval is the boundary of normal head movement.

During his earlier intake studies, he showed a delay in saccades on VNG, as shown in Figure 4A. He also showed a loss of balance capacity on his posturography in Figure 4B. These studies contributed to his diagnosis of vertigo of central origin. He was undergoing balance rehabilitation when he developed symptoms of disruption of his prior stomach banding, necessitating surgical intervention. He survived a second emergency surgery due to complications from this repair of his stomach banding. He returned to his balance therapy.

Case 5

**Figure 5:**
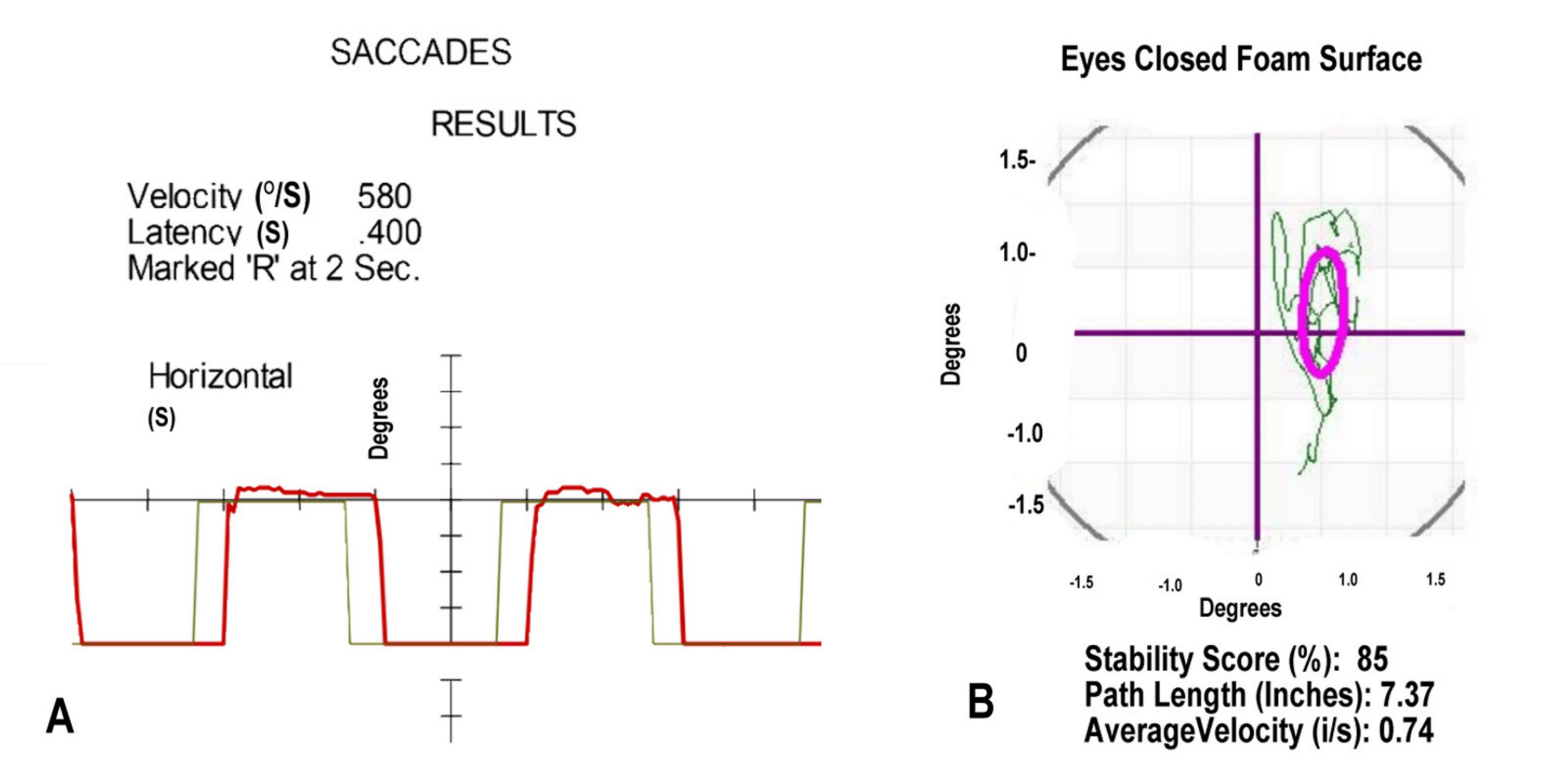
Video Nystagmography and Computerized Posturography for Case 5 (A) Saccades (red line), measured with a moving light, show a prolonged latency of 0.400 seconds. (B) Head movement during posturography (green line) shows significant head movement outside the norm (purple oval) while standing on a foam surface with eyes closed.

A 50-year-old female presented four weeks after her motor vehicle accident, complaining of dizziness, headaches, and difficulty writing with her right hand. She reported pain in her hand while using a pen, as well as increasing incoordination one month after her accident. Measures of extended wrist rotation are shown in Table 1. Concurrently, she showed abnormal VNG and posturography, as shown in Figure 5.

Statistical results

The simple paired “t” test to compare groups, and the ANOVA performed on the entire population, showed a statistically significant difference between the separate extended external and internal wrist rotations before and after treatment. The combined measures also show evidence of treatment effect (Table 2). Table 1 shows the raw data from each case before and after treatment.

**Table 2:** Paired Statistical Analysis of Case Series Treatment Each case subgroup contains measures of extended wrist rotation before and after treatment with the commercially sourced isomerized potassium linoleate. The complete list of measures is shown in Table 1. Separate (paired t-test) and combined ANOVA show supination (external rotation) and pronation (internal rotation) as statistically distinct groups and a significant treatment effect. The p- and F-values for paired t-tests and ANOVA are recorded for descriptive purposes. For a case series of 5, having significance in the difference in means is of interest, but has little predictive value. ANOVA, Analysis of Variance

External Rotation	Internal Rotation	Combined Rotations, ANOVA
p = 3.587 x 10^-4^	p = 1.522 x 10^-2^	p = 8.815 x 10^-8^; F = 328

## Discussion

Isomerized KCLA has a cross-kingdom bactericidal and fungicidal capacity sufficient to satisfy the same Environmental Protection Agency (EPA) standards for hospital disinfection used for quaternary amines and hypochlorites [12]. The commercial cosmetic isomerized KCLA has sufficient detergent capacity, as well as antibacterial and antifungal biocidal capacity across kingdoms, to effect reductions in major components of the skin biome. With its known cross-kingdom antimicrobial efficacy, topical application reduced rigidity associated with neurodegenerative disease [13]. The finding of post-traumatic rigidity with extended wrist rotation impairment led to the application of KCLA to the skin to determine its effect on rigidity evolving after trauma.

Isomerized KCLA has a fatty acid correlated with the common name, rumenic acid. This references its presence in the rumens of mammals. That KCLA may serve a role as a biomic regulator, long hidden in gut homeostasis, provides an intriguing background to this case series. Humans have an established blood level of conjugated linoleic acid from animals that “chew the cud.” The human microbial biome, including skin, maintains homeostasis through chemical communications with QSAs. The defining purpose of these QSAs is to initiate group invasive behaviors when the concentration of the specific QSA reaches a “quorum.” However, these QSAs can have direct effects on the host. In addition to lactones, Pseudomonas secretes a quinolone (Pseudomonas Quorum Sensing Agent, or PQSA) that suppresses the immune system. In one brain slice study [10], topical PQSA disordered neuronal function. Intraperitoneal injection of PQSA caused a stupor lasting hours.

Inflammatory cytokines applied to sensory axons can initiate antidromic discharge. The antidromic signaling may cause the afferent to discharge neuroinflammatory agents such as substance P, calcitonin gene-related peptide, glutamate, adenosine triphosphate (ATP), and neurokinin A [14]. The skin has complex and dense interactions with the interneurons within the spinal cord to coordinate the movement of the arms and legs. Inflammation-induced antidromic neuronal discharge can alter muscle tone by discharging ATP, glutamate, neurotensin A, substance P, and/or calcitonin-related gene peptides [15].

Consider *Malassezia* as a potential participant in this response, because it is the most abundant skin microbe. It has clear access to the nerves within hair follicles and skin to initiate orthodromic and antidromic signals through inflammatory cytokines. For the potential benefit of *Malassezia*, the afferent synaptosomes contain nutrients usable by *Malassezia*. The feeding of *Malassezia* by neural products could, in turn, aid further activation of quorum-sensing behavior while contributing to the inflammatory state. During inflammation, an orthodromic impulse could activate a primary sensory arc to the anterior horn motor gamma efferent neuron to increase the tone on the muscle spindle, which in turn could cause involuntary motor contraction. This contraction would not follow the rhythmic contraction and relaxation characteristic of voluntary muscles. That portion of the muscle in sustained tetany would never relax to allow the lymph vessels with valves to fill and pump the lymph into the venous system [16]. The sustained tetany would not only create rigidity; it would obstruct the flow of lymph fluid.

That our microbiome has constant interaction with neurons transporting molecules means the content of our spinal cords changes with the contents of our skin microbiome. Indeed, gold nanoparticles applied to the skin of a rat travel to their dorsal root ganglia within six hours [17].

Tetany and tenderness of the supinator muscle, with loss of extended wrist range of motion, represent a novel clinical constellation to re-think evolving rigidity in trauma. The onset of rigidity with focal muscular tetany satisfies the requirement of one clinical finding to diagnose Parkinsonism (two are needed for the clinical diagnosis of PD). In this case series, physical therapy improved symptoms of balance impairments and dizziness, along with mobility, but did not alter the tetany within the supinator. Traumatic brain injuries have been linked to an increased likelihood of neurodegenerative disorders associated with rigidity [3,4]. The UPDRS incorporates extended wrist rotation as a measure of rigidity [2].

That a simple application of topical isomerized KCLA rapidly reduced the focal rigidity and tetany of the supinator muscle creates many questions. The absence of the clinical findings for neural compressive injuries points to something distinct, not an overuse syndrome. Yet, untreated, persisting use of the arm in the presence of tetany would favor the precipitation of common overuse injuries involving neural compression.

The link between *Malassezia* and rigidity has caused many to speculate a causal relationship to neurodegenerative disease, and specifically PD [18]. That KCLA has antimicrobial capacity against *Malassezia* supports the prospect that *Malassezia*, or some combination of fungi-bacteria, may play an active role in focal tetany and rigidity [19].

Limitations of the current case report series

This case report series lacks microbiological support. The above speculative conclusions have no direct “cause and effect” experimental or clinical data linking the observed clinical supinator tetany to a change in commensal behavior in the skin. The reversal of the tetany or rigidity of the supinator muscle with topical application of a solution of isomerized KCLA within minutes “points to,” but does not prove, the skin's microbiome contributes to rigidity or tetany. This case series represents the earliest phase of a novel medical finding that needs confirmation by independent investigators. The study does not limit or imply applicability beyond this patient population.

## Conclusions

A topical cosmetic cleanser with a proprietary blend of isomerized KCLA, explored as a salve, reduces supinator tetany or rigidity within minutes in all five cases experiencing painful supinator tetany or rigidity. This case series provides objective clinical findings that open the door to further exploration of topical isomerized KCLA to reduce clinical rigidity.
